# Maternal Experiences with Exclusive Pumping—An Online Survey

**DOI:** 10.3390/healthcare14101361

**Published:** 2026-05-15

**Authors:** Zoya Gridneva, Jacki L. McEachran, Demelza J. Ireland, Sharon L. Perrella, Donna T. Geddes

**Affiliations:** 1 School of Molecular Sciences, The University of Western Australia, Crawley, WA 6009, Australia; jacki.mceachran@uwa.edu.au (J.L.M.); sharon.perrella@uwa.edu.au (S.L.P.); donna.geddes@uwa.edu.au (D.T.G.); 2 ABREAST Network, Perth, WA 6000, Australia; 3 UWA Centre for Human Lactation Research and Translation, Crawley, WA 6009, Australia; 4 School of Biomedical Sciences, The University of Western Australia, Crawley, WA 6009, Australia; demelza.ireland@uwa.edu.au

**Keywords:** exclusive pumping, lactation, breastfeeding, human milk, breast pumping, breast expression, electric breast pump

## Abstract

**Highlights:**

**What are the main findings?**

- Most exclusively pumping women had originally intended to partially or fully breastfeed, with only 5% reporting that exclusive pumping was their original plan.
- Exclusively pumping women reported a lack of resources and information to guide pumping logistics, milk storage and feeding.

**What are the implications of the main findings?**

- Targeted education for health professionals on support and information needed by exclusively pumping women is recommended.
- Tailored, evidence-based guidelines will help to support exclusively pumping women and improve maternal well-being.

**Abstract:**

**Background/Objectives**: The prevalence of women who exclusively pump (EP) their breast milk to feed their infants is increasing; however, this group is underrepresented in research. This study aimed to examine maternal and infant characteristics and the experiences of EP women. **Methods**: An online survey explored the experiences and characteristics of EP women with <24-month-old infants. Quantitative data included demographics and maternal and infant characteristics; qualitative data included perspectives on how support for EP women can be improved. **Results**: The survey of 195 EP women revealed that while most had intended to exclusively breastfeed (50%) or breastfeed and pump (26%) their milk for an average of 12 months, the average time of EP cessation was 6 months postpartum. Compared with the general population, EP women had higher rates of pregnancy complications (*p* < 0.001) and lactation/breastfeeding challenges (*p* < 0.001). Themes developed from the qualitative data relating to how health professionals/support people could better assist EP women were: ‘Respect for the EP Journey’, ‘EP Information and Logistical Needs’ and ‘Mental and Physical Load’. **Conclusions**: Most EP women had originally intended to breastfeed but utilised EP because of latching issues, breast refusal and/or neonatal unit admission. They face unique challenges associated with EP, yet current professional acceptance and support are lacking. Targeted education for health professionals on EP is needed so that they can better support women with tailored, evidence-based guidelines aimed at extending lactation and improving maternal well-being.

## 1. Introduction

Breast milk is composed of nutrients and bioactive compounds that provide conditionally complete nutrition for infants, and is the optimal primary food source for infants up to 6 months old [1]. The World Health Organization (WHO) describes exclusive breastfeeding as the provision of exclusive human milk, whether that be directly from the breast, expressed breast milk (EBM) or donor milk, with no supplementation with other liquids or solids, with the exception of oral vitamins and medication [2]. Despite multiple breastfeeding benefits [3,4,5,6,7], global breastfeeding rates consistently fall short of WHO recommendations, with only 48% of infants under 6 months of age exclusively breastfed [8]. Many women cease breastfeeding earlier than recommended due to nipple pain, perceived/true low milk supply, and infant oral anomalies [9,10]. Caesarean section, preterm birth and neonatal intensive care unit (NICU) admission are also associated with reduced breastfeeding duration [11,12,13], along with societal barriers, such as returning to paid work and lack of breastfeeding education [8,10,14].

Breast milk expression is a method of removing milk from the breast that does not involve the infant latching on the breast. Milk is removed by hand expression with the application of pressure to the milk ducts, or by manual or electric breast pumps that apply a vacuum [15]. Milk expression with a pump, or ‘pumping’, is increasingly common due to improved technology, women returning to paid employment, breastfeeding difficulties and the desire to share infant feeding responsibilities [15,16,17,18], particularly during the early months of lactation [19]. One American study stated that 85% of women express milk within the first four months postpartum [20], whilst a recent Australian study reported that 47% of women pumped at least once in 24 h at 2–3 months postpartum [21]. Evidence of the impact of pumping on maternal and infant health outcomes is still extremely limited. Frequent pumping can cause nipple trauma, reduce mother–infant skin-to-skin contact, and increase workload due to the additional time associated with cleaning of pumping equipment [14,18]. EBM is typically delivered via a bottle, and bottle feeding has been linked to reduced self-regulation of milk intake [22] and accelerated weight gain [23].

Exclusive pumping (EP) involves providing breast milk for an infant only by feeding EBM [15]. While EP is often initiated due to NICU admission or latch issues, many mothers persist with EP when direct breastfeeding is not possible as they value the benefits of breast milk for their infants [15,18,24]. The prevalence of EP varies, with geographically limited reports indicating rates of 7% in Western Australia [21] and 6–14% in the USA [25]. Higher EP prevalence is seen in Asia, with rates of 17% in Singapore [17], where EBM feeding is common among Chinese parents [26], 20% in Hong Kong [14] and 23% in China [27]. The highest EP prevalence was recently reported for ethnic Chinese parents living in Malaysia, with rates of 39–62% [28], and is partially contributed to Chinese parenting styles [29]. EP is more common among women with infant latch difficulties, flat or inverted nipples, mastitis, breast refusal, preterm or caesarean births, NICU admission and multiple births [15,18,21,27]. Other contributing maternal factors include primiparity, lack of breastfeeding experience and shorter maternity leave [15,21].

A recent small study confirmed that with effective electric pumps, EP women can maintain normal milk production [21] (defined as ≥600 g/24 h [30]); however, not all women have access to such pumps. EP has been associated with shorter overall breastfeeding duration and earlier formula introduction compared with combined pumping and direct breastfeeding [18,25,31]. In Singapore and China, the duration of breastfeeding with EP ranged between 5 and 7 months, while in the USA it was 2 months [15].

Previous EP-related research is extremely limited, with a recent scoping review identifying only seven papers on EP [18], which focused predominantly on the experiences of EP women [15,18,32,33]. The time and work associated with EP, including pumping and feeding, cleaning and maintenance of pumping equipment, and EBM storage, are commonly reported challenges [15,18]. EP is time-intensive, and has been described as burdensome and similar to a full-time job; however, EP women report that, with time and experience, EP becomes more feasible [15]. Additionally, many EP women report a sense of failure, grief and shame due to their inability to directly breastfeed [32]. Further, guidance on pumping practices for EP is inconsistent [18] with EP women reporting that information on pumping frequency, pumping session duration, breast shield (flange) sizing and maintaining supply are key at the beginning of their EP journey. However, due to the lack of EP-specific guidance, many women turn to informal information sources such as online support groups, where misinformation is common [33].

The growing prevalence of EP, coupled with EP women’s reliance on informal sources of information, highlights the need for research to inform clinical guidelines that health professionals can use to better support EP women. Additionally, as breastfeeding outcomes differ by parity [34], the experiences and satisfaction with clinical care and support of EP women may also be different. Further, due to the differing expectations of sick/preterm infants’ breastfeeding abilities [35], a comparison of the experiences of EP women whose infants were born preterm (<37 weeks’ birth gestation) and had a NICU admission with those of women with healthy term infants may further aid the care and support of EP women. This exploratory study adopted a mixed-methods approach and used an online survey to collect data on EP women’s demographics, characteristics and experiences, as well as their pumping and feeding dynamics, aiming to provide insight into their breastfeeding journey.

## 2. Materials and Methods

### 2.1. Study Design and Participants

EP women up to 24 months postpartum completed an online anonymous 85-item survey using the secure web-based software platform designed to support data capture for research studies, Research Electronic Data Capture (REDCap, version 15.5.23), hosted at The University of Western Australia [36,37]. The survey was co-designed with experienced lactation and maternity care professionals, and developed with engagement from consumers to ensure validity and relevance. Survey responses captured participants’ demographic data and characteristics, including breastfeeding intentions and information seeking during pregnancy, perinatal, health and breastfeeding characteristics, lactation and EP challenges, accessed breastfeeding services and experience of care by health professionals, as well as pumping and feeding dynamics and logistics (Supplementary Materials S1).

Quantitative items collected participants’ demographics, maternal and infant characteristics and pumping and feeding logistics. An open-ended question allowed for the collection of qualitative data on how support for EP women can be improved. The typed responses had no word limit. Participants’ satisfaction with the accessed sources of educational and professional support, as well as support people’s responses to their EP journey, was rated using a Likert scale.

Participants were recruited through online social media posts on Facebook groups which target women that have experience of EP in Australia, New Zealand, the USA, the UK and France. The Facebook groups included online peer-support groups for EP women and for parents of preterm/NICU infants. Survey inclusion criteria were English-speaking women ≥ 18 years old who had exclusively expressed their milk to feed their infant(s) who were <24 months old. The reliability of maternal recall of birth and breastfeeding events within 24 months postpartum is generally considered to be high [38,39].

For the survey participants, EP was defined as follows: “For this study exclusive pumping (EP) is defined as only feeding the baby pumped/expressed breast milk. That is, the baby does not drink breast milk directly from the breast. A mother is still considered to exclusively pump if the baby also fed formula. Please note that the term ‘pumping’ refers to any form of breast milk expression including hand expression, manual or electric pumping. You are welcome to answer the survey questions if you are currently exclusively pumping or have exclusively pumped in the past and your baby is under 24 months of age.” (Supplementary Materials S1.)

The study was approval by The University of Western Australia Human Research Ethics Committee (2025/ET000304). Participants completed a digital participant information and consent form and were aware of their right to withdraw from the survey at any time with no consequence and assured of confidentiality and privacy.

This paper focuses on participants’ demographics and maternal and infant characteristics, as well as lactation and EP challenges and support for EP women.

### 2.2. Statistical Analysis

For the quantitative component of the study, a sample size of 90 was determined using the ‘F tests—Linear multiple regression: Fixed model: R^2^ increase option’ in G*Power 3.1 [40]. With one predictor, a small effect size (Cohen’s *f*^2^ below 0.1), and an alpha level of 0.05, a total sample size of 90 participants was determined to achieve a power of 0.80 [41]. The target sample size was increased to 100 to account for missing data. For qualitative research, a sample of *n* ≥ 30 is generally considered adequate to achieve data sufficiency [42].

Continuous data were assessed for normal distribution using the Normality Test (Shapiro–Wilk) and Q-Q plots, and described as mean ± standard deviation (SD) for normally distributed data or median [Q1, Q3] for skewed data. Categorical data were described by frequencies/counts and percentages.

Student’s *t*-test, the Chi-square test or Fischer’s exact test were used as appropriate to compare variables of women with term infants that did not have a neonatal nursery and/or NICU (neonatal unit) admission (healthy term (HT) group) and those whose infants were born preterm (<37 weeks’ birth gestation) and/or had a neonatal unit admission (sick/preterm (SP) group). We anticipated that the different groups’ access to information, experiences and satisfaction with clinical care and support may be different due to the differing expectations of sick/preterm infants’ breastfeeding abilities [35]. Additionally, we conducted a comparison of primiparous and multiparous EP women as breastfeeding outcomes differ by parity [34]; only significant differences are presented for this comparison.

The Chi-square test was also used to compare EP women’s health characteristics and lactation difficulty frequencies (Australian only, *n* = 180) with the available published general and breastfeeding population data reported in Australia [43,44,45,46,47,48,49].

Missing data were addressed using available case analysis (pairwise deletion) to have a higher efficiency, avoid bias and loss of precision and to preserve the sample sizes [50]. Results of this exploratory study were considered statistically significant when *p* < 0.05; adjustments for multiplicity have not been conducted and caution should be exercised when interpreting *p*-values. All quantitative analysis was performed using Excel and/or Jamovi software (Version 2.6.25).

### 2.3. Qualitative Analysis

Qualitative responses to the open-ended question of ‘How can health professionals and support people better support exclusively pumping women?’ were analysed using thematic analysis, as per the Braun and Clarke framework [51]. The participant responses were independently read by two researchers (LH-SH and SLP) multiple times to ensure familiarity with the data. One researcher (LH-SH) had no background in breastfeeding support, and the other (SLP) was an experienced midwife and international board-certified lactation consultant. An inductive (rather than reflexive) thematic analysis was chosen as it is best suited to describing maternal experiences of EP, allowing themes to emerge from participants’ voices rather than being influenced by the researchers’ active and subjective roles in constructing meaning from the data.

The researchers individually read and re-read the data, noting initial ideas, impressions, and patterns, while also documenting any personal assumptions or pre-conceptions. Code words or phrases that reflected the meaning of the responses were recorded, compared and discussed by the researchers, using an iterative process. Any discrepancies in coding were resolved through discussion, including consideration of any biases, until a consensus was reached. Broad patterns or themes were identified from the codes and further refined into subthemes with a thematic map constructed to represent the participants’ experiences and perspectives. Finally, key quotes from participants were selected based on their effectiveness in representing a theme or illustrating a subtheme. These were then included in the reporting of results. Credibility was ensured by maintaining detailed records of the coding process, use of researcher triangulation and reflexivity. Trustworthiness was ensured by confirming with selected participants that the study’s findings accurately reflected the qualitative data, and by checking with health professionals that the findings were applicable to practice.

## 3. Results

### 3.1. Participants’ Demographics and Expressing Status

**Figure 1:**
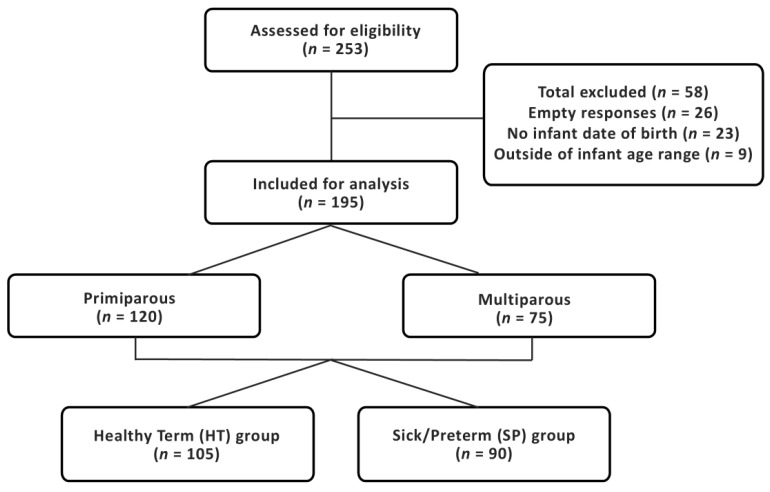
Study recruitment flow chart. HT, healthy term group—women with term infants that did not have a neonatal unit admission; SP, sick/preterm group—women with infants that were born preterm (<37 weeks’ birth gestation) and/or had a neonatal unit admission.

Participants were recruited from April 2025 to September 2025. Of the 253 responses received, *n* = 195 met the study criteria, of which 120 were primiparous and 90 had infants that were born preterm (<37 weeks’ birth gestation) and/or had a neonatal unit admission (SP group; Figure 1). Most women (58%) were currently EP at the time of the survey (Table 1). Of those women not currently EP, the average infant age at EP cessation was 6 [4.0, 9.0] months; the main reason for EP cessation was that it is too difficult to pump and take care of an infant (42%). The majority of women who completed the survey were tertiary educated (66%), married or in a de facto relationship (93%) and lived in Australia (92%) (Table 2). Although we had attempted a multinational survey, the response rate outside Australia was very low (*n* = 15); given the responses from participants residing outside Australia were not noticeably different from those from Australia, it was decided to retain them in our data for analysis. EP women had overweight body mass index (BMI) (29.1 [24.8, 32.8] kg/m^2^). There were no significant differences between primiparous and multiparous women in the infant age at the time of participation in survey, in demographics, BMI, or in infant age at cessation of EP and reasons for EP cessation. Women from the SP group were more likely to be Australian (97% vs. 89%, *p* = 0.034) and primiparous (67% vs. 57%, *p* = 0.037) (Table 1 and Table 2) than women from the HT group and had lower pre-pregnancy (26.7 [22.5, 31.0] vs. 28.7 [25.1, 33.1], *p* = 0.001) and current BMI (27.5 [23.3, 32.1] vs. 30.1 [26.3, 33.9], *p* = 0.001) (Table 2).

### 3.2. Breastfeeding Intentions and Information Seeking During Pregnancy

During pregnancy, half of the EP women (50%) planned to breastfeed exclusively and most intended to breastfeed for 12 months (Table 3). Multiparous women were more likely to plan to combine breastfeeding and pumping than primiparous women (26/75 (35%) vs. 25/120 (21%), *p* = 0.030). The majority of women accessed breastfeeding information from their midwife/obstetric nurse (47%) and online/social media sources (43%) (Table 3). More primiparous than multiparous women accessed breastfeeding information from their midwife/obstetric nurse (64/120 (53%) vs. 27/75 (36%), *p* = 0.018), obstetrician (22/120 (18%) vs. 6/75 (8%), *p* = 0.045) and breastfeeding classes (39/120 (33%) vs. 9/75 (12%), *p* = 0.001). Women from the SP group were more likely to plan formula feeding (4% vs. 0%, *p* = 0.044), not to access breastfeeding information (36% vs. 11%, *p* < 0.001) and access less information from online and social media sources (29% vs. 55%, *p* < 0.001) during pregnancy than women from the HT group (Table 3).

### 3.3. Participants’ Perinatal, Health and Breastfeeding Characteristics

Most women had a singleton birth (93%) and reported at least one pregnancy complication (65%) (Table 4). More primiparous women had hypertension during pregnancy than multiparous women (26/120 (22%) vs. 8/75 (11%), *p* = 0.049). Further, women from the SP group had more multiple births (14% vs. 1%, *p* < 0.001) and various pregnancy complications, including foetal growth restriction (31% vs. 6%, *p* < 0.001), preeclampsia (26% vs. 6%, *p* < 0.001), placental insufficiency (16% vs. 2%, *p* < 0.001) and other (32% vs. 12%, *p* < 0.001). The majority of women (75%) reported that their breasts grew by one bra cup size or more and that they perceived that their breast density changed (81%) during pregnancy (Table 4).

Multiparous women were more likely to have no pre-existing health conditions than primiparous women (42/75 (56%) vs. 42/120 (35%), *p* = 0.004) with primiparous women more likely to report anxiety (45/120 (38%) vs. 17/75 (23%), *p* = 0.030), depression (30/120 (25%) vs. 9/75 (12%), *p* = 0.027), and less breast cysts (1/120 (1%, vs. 8/75 (11%), *p* = 0.002). Women from the SP group had more fertility issues (23% vs. 10%, *p* = 0.009) compared with women from the HT group (Table 5).

Approximately a third of infants (31%) were born preterm and almost half of infants (43%) were admitted to the neonatal unit (Table 6). Type of birth varied by parity, with multiparous women more likely to have an unassisted vaginal birth (31/75 (41%) vs. 32/120 (27%), *p* = 0.033) or planned caesarean section (24/75 (32%) vs. 22/120 (18%), *p* = 0.029). Primiparous women were more likely to have vacuum-assisted vaginal birth (7/120 (6%) vs. 0/75 (0%), *p* = 0.045), forceps-assisted vaginal birth (7/120 (6%) vs. 0/75 (0%), *p* = 0.045) and unplanned caesarean section (52/120 (43%) vs. 20/75 (27%), *p* = 0.019). Women from the SP group were less likely to have an unassisted vaginal birth (13% vs. 49%, *p* < 0.001) and more likely to have unplanned caesarean section (59% vs. 18%, *p* < 0.001) than women from the HT group (Table 6).

Birth gestation did not differ by parity (primiparous: 38.3 [34.4, 39.3], multiparous: 38.7 [37.0, 39.4]; *p* = 0.23). Ninety-two percent of infants in the SP group were admitted to the neonatal unit and 44% had at least one health condition (*p* < 0.001).

Most women (78%) attempted direct breastfeeding before starting EP; however, women from the SP group were almost twice less likely to attempt it (56% vs. 97%, *p* < 0.001) (Table 6). The duration of attempting direct breastfeeding was not different between the HT and SP groups. However, multiparous women were more likely to still attempt to directly breastfeed at the time of the survey than primiparous women (15/56 (27%) vs. 12/96 (13%), *p* = 0.026) and their infants were more likely to have latched well during the first week after birth (18/56 (32%) vs. 13/96 (14%), *p* = 0.006). Further, infants of women from the SP group were less likely to latch well compared with infants from the HT group (62% vs. 39%, *p* = 0.008).

EBM was the most common source of the infant diet in the first week after birth (85%). Infant diet during the first week did not differ by parity, but infants from the SP group were more likely to receive EBM (93% vs. 77%, *p* = 0.002), donor milk (18% vs. 2%, *p* < 0.001) and less breast milk directly from the breast (21% vs. 67%, *p* < 0.001) (Table 6).

### 3.4. Lactation Challenges and Accessed Breastfeeding Services

All EP women (100%) experienced lactation challenges (3.2 ± 1.7 challenges on average), and almost three quarters (74%) experienced latching issues (Table 7). Whilst there was no difference by parity in terms of lactation challenges, women from the SP group less frequently reported latching issues (66% vs. 81%, *p* = 0.015) and damaged/painful nipples from breastfeeding (26% vs. 54%, *p* < 0.001) compared to women from the HT group.

**Figure 2:**
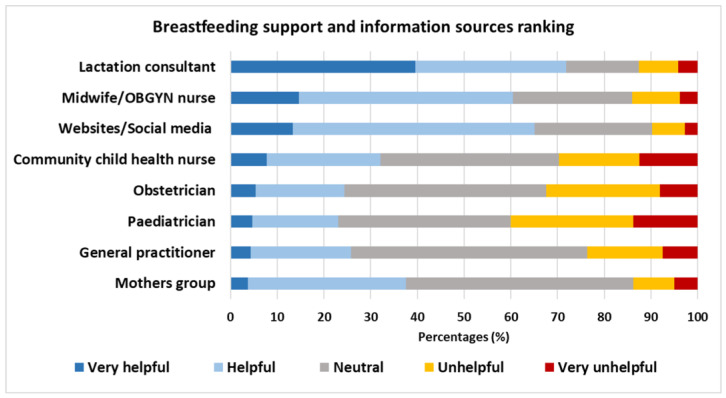
Perceived helpfulness of support services and/or information sources accessed for help with trying to breastfeed. Data presented as %; OBGYN, obstetrics and gynaecology.

Most EP women (81%) accessed a lactation consultant or a midwife (53%) when they experienced breastfeeding challenges (Table 7). Very few women did not access any breastfeeding services, and this was more common amongst multiparous women than primiparous women (6/75 (8%) vs. 3/120 (3%), *p* = 0.024), who were also less likely to access breastfeeding websites (25/75 (33%) vs. 63/120 (53%), *p* = 0.009). Women from the SP group were less likely to access breastfeeding information at mothers’ groups (13% vs. 28%, *p* = 0.015) than women from the HT group (Table 7). When asked to rate the helpfulness of breastfeeding support and information sources, women were more likely to rate lactation consultants as very helpful (40%), followed by midwives (15%) and websites and social media (13%); paediatricians (14%) and community child health nurses (13%) were more likely to be rated as very unhelpful (Figure 2).

Multiparous women were more likely to have learnt about EP prior to this pregnancy than primiparous women (55/75 (73%) vs. 28/120 (23%), *p* < 0.001) and to be unsure about it (6/75 (8%) vs. 1/120 (1%), *p* = 0.014), whilst primiparous women were more likely to have learnt about EP during this pregnancy (24/120 (20%) vs. 2/75 (3%), *p* < 0.001) or after the birth of this infant (67/120 (56%) vs. 12/75 (16%), *p* < 0.001). Women in the SP group were less likely to learn about EP before this pregnancy (34% vs. 50%, *p* = 0.034) than women from the HT group (Table 8).

Commonly reported factors leading to EP included latching issues (62%), breast refusal (34%) and/or neonatal unit admission (33%) (Table 8). Women from the SP group were more likely to report having birthed multiples (10% vs. 1%, *p* = 0.006) and infant health conditions (29% vs. 15%, *p* = 0.021) as reasons leading to EP. They were also less likely to report infant latching issues (48% vs. 74%, *p* < 0.001), breast refusal (24% vs. 42%, *p* = 0.010) and slow infant weight gain (1% vs. 10%, *p* = 0.012), as well as pain during direct breastfeeding (12% vs. 36%, *p* < 0.001), as reasons leading to EP than women from the HT group (Table 8).

The main challenges experienced with EP were time constraints with pumping (85%) and managing pumping around infant care (83%), as well as having to pump at night (72%). Primiparous women were more likely than multiparous women to report challenges relating to a lack of knowledge (46/120 (38%) vs. 17/75 (23%), *p* = 0.023) and EBM storage (52/120 (43%) vs. 22/75 (29%), *p* = 0.04995). Women from the SP group were less likely to report managing pumping around infant care (76% vs. 90%, *p* = 0.010) and discomfort caused by pumping (48% vs. 65%, *p* = 0.017) as EP challenges compared to women from the HT group (Table 8).

**Figure 3:**
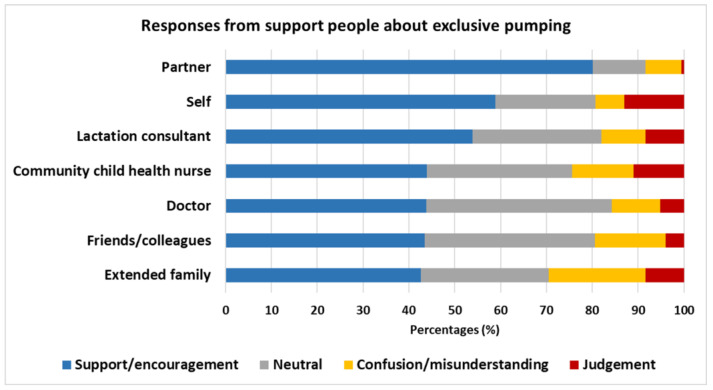
Responses to exclusive pumping journey by support people. Data are presented as %.

When asked to describe the responses EP women received from their support persons about EP, women rated their partner as the ones with the most support and encouragement (80%), followed by self (59%) and lactation consultants (54%) (Figure 3). However, EP women also rated themselves as the most judgmental (13%), followed by community child health nurses (11%) and extended family (8%), whilst their partners were rated as the least judgmental (0.5%). Extended family members were also reported as the most confused with/not understanding the EP journey (21%).

### 3.5. Comparison with the General Population

Australian EP women in this cohort (*n* = 180) had higher rates of caesarean section birth than the general Australian population (62% vs. 39%, *p* < 0.001); higher rates of pregnancy complications and infant health conditions, including gestational hypertension (17% vs. 3%, *p* < 0.001), preterm birth (32% vs. 8%, *p* < 0.001) and neonatal unit admission (45% vs. 18%, *p* < 0.001); and higher rates of anxiety and depression (50% vs. 16%, *p* < 0.001) (Table 9). These EP women were also more likely to experience higher rates of nipple pain (78% vs. 36%, *p* < 0.001) and mastitis (26% vs. 17%, *p* = 0.004) (Table 9).

### 3.6. Qualitative Findings

**Figure 4:**
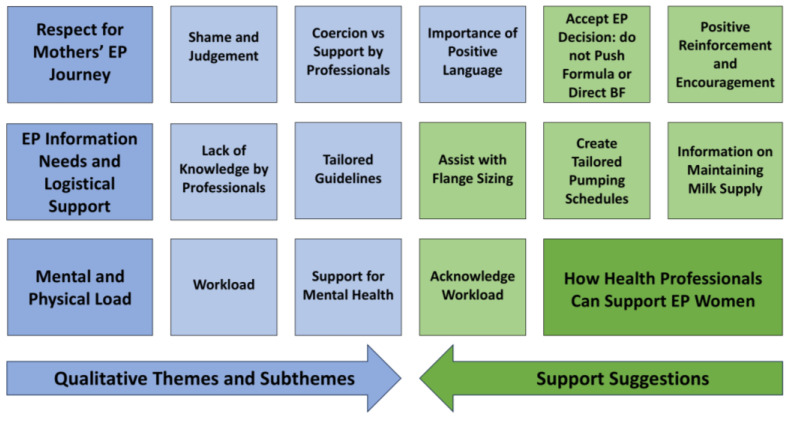
Qualitative themes (dark blue boxes) and subthemes (light blue boxes) and suggestions on how healthcare professionals can support EP mothers (green boxes).

Most participants (*n* = 183 (94%)) provided qualitative data for the open-ended question ‘How can health professionals and support people better support exclusively pumping women?’ An inductive thematic analysis identified three main themes: ‘Respect for Mothers’ EP Journey’, ‘EP Information Needs and Logistical Support’, and ‘Mental and Physical Load’ (Figure 4). Quotes are reported below to further illustrate the themes, with codes used to identify each participant’s parity, i.e., (P)—primiparous and (M)—multiparous. Qualitative findings for how health professionals can better support EP mothers are summarised in Figure 4.

#### 3.6.1. Theme 1. Respect for Mothers’ EP Journey

This theme explored how the reactions of health professionals and support people towards a woman’s decision to EP can promote or damage her morale.

- Subtheme 1 (Shame and Judgement) represented how health professionals can perpetuate feelings of inadequacy and shame due to harmful narratives associated with EP being seen as inferior to direct breastfeeding. Importantly, consistent acknowledgement that EP is breastfeeding was seen to reduce feelings of failure:

*“Don’t just put “bottle feeding” down, specify it’s my milk! I work hard for that.”*

(M)

*“I think one of the biggest things support people and health professionals could do is help reduce the shame that often surrounds not feeding directly from the breast. There’s still such a strong narrative that “real” breastfeeding only means feeding at the breast, which can leave exclusive pumpers feeling like they’re failing or missing out. It would help so much if professionals consistently acknowledged (and reinforced to families) that pumping is breastfeeding—that it’s valid, valuable, and just as nourishing.”*

(P)

Women cited that a lack of understanding of EP by family members led to judgement:

*“Family members don’t understand pumping. They just see you giving a bottle and judge you straight away.”*

(P)

*“Wish husband had been more supportive—to him he was behind whatever I wanted but when I struggled with EP he would always be like “we can switch to formula if you want.”*

(P)

- Subtheme 2 (Coercion versus Support by Professionals) highlighted that many health professionals did not provide individualised care, with women feeling like their personal needs and wants were not being heard or acknowledged. This resulted in many women feeling coerced into using a feeding method preferred by the care provider against their own wishes. Coercion included pressure to switch to formula or to persist in trying to directly breastfeed despite the woman not being able or no longer wanting to:

*“Some community nurses I saw were really pushy about trying to breastfeed even though I knew she would struggle to cope with my fast flow and short nipples. All my kids have. It was frustrating to have them push it on me as though they had the answer to my problems even though I knew from experience that my kids need time and to grow a little. I wanted to breastfeed rather than exclusively pumping so if it was possible I would’ve already been breastfeeding.”*

(M)

*“Formula is thrown around as the easier better option I was told by several people friends professionals whatever that formula could just solve all our issues and bub would be ok. Well, no bub wouldn’t be ok drinking powdered chemical processed junk with water, women aren’t supported instead told to do what’s easier and best for mom and lying and saying it’s best for bub, there needs to be better education classes on flanges, pump schedules, routines, access to affordable pumps* etc.”

(P)

Women suggested that aligning health professionals’ advice with the goals of the individual would allow mothers to feel better supported:

*“Acknowledge that it’s a valid way of feeding the baby, ask what we need to make it easier, ask how we are going/coping with it, ask about problems we’ve run into, don’t suggest formula instead just ask us what our goals are.”*

(P)

- Subtheme 3 (Importance of Positive Language) addressed the impact of encouraging and supportive language on EP women’s confidence and self-esteem. Women felt validated when health professionals made direct statements of encouragement:

*“Unfortunately I had lost a lot confidence in pumping after a second bout of mastitis (around 10 weeks) and a lactation adviser I saw simply said how great it was that I had come so far and had not given up—and it was such a needed boost!”*

(M)

Health professionals’ reference to EP as breastfeeding promoted feelings of approval:

*“When pediatrician asked about feeding I said I was exclusively pumping—her response was “so breastfeeding” made me feel validated.”*

(P)

#### 3.6.2. Theme 2. EP Information and Logistical Support

- Subtheme 4 (Lack of Knowledge by Professionals) highlighted the lack of EP-specific knowledge among health professionals. Many women stated that a lack of evidence-based, practical guidelines meant that contradictory advice was given, further contributing to the stress of EP: *“More pumping education in the midwife/health nurse space. A lot provide outdated or conflicting advice.”* (M)

*“There is very little information for people who express exclusively. This means that even supportive people don’t quite know what to say.”*

(M)

When health professionals could not provide adequate information, women often turned to social media. A common gap in practical support was the lactation consultants’ lack of knowledge on breast pump flange sizing. As a result, many women reported having to search for flange size information online:

*“Community maternal child health nurses and lactation consults should be able to assist pumping mothers. None measured my nipples to ensure correct flange size, I had to work this out via social media channels.”*

(P)

- Subtheme 5 (Tailored Guidelines) focused on the importance of tailored guidelines for EP:

*“Provide more information on exclusively pumping—how often to pump, types of pumps, flange sizes, sterilisation requirements for equipment, recommendations around how often to clean the pump and parts.”*

(P)

*“Knowledge around specifics* e.g., *importance of correct flange size and how to measure. Blanket adherence to one recommendation re: timed breastmilk safety (*e.g., *sometimes told breastmilk is ok out of fridge for up to 8 h, other times told only 2 h)”.*

(M)

Many EP women emphasised the need for personalised advice that suits the unique needs of the individual:

*“Health professionals could better support us by giving practical help and resources specifically for pumping: creating personalised plans, helping navigate supply issues, and recognising the mental load involved. Even small things like acknowledging how hard and impressive it is to keep going would make a huge difference. Overall, more empathy, validation, and tailored guidance for pumping parents would go a long way.”*

(P)

#### 3.6.3. Theme 3. Mental and Physical Load

- Subtheme 6 (Workload) highlighted a commonly identified challenge of EP, the physical workload and time demand that EP places on women during the vulnerable postpartum time. The additional work involved with EP included time spent pumping and then feeding EBM to the infant, cleaning and maintaining pumping equipment, and handling and storing EBM:

*“I think there needs to be much more understanding of the sheer work involved. Pumping is incredibly demanding and stressful, particularly long term—it’s not just feeding the baby, but also managing sterilisation, scheduling sessions, washing parts, storing milk, and often doing it all alone in the middle of the night. It’s physically and emotionally exhausting.”*

(P)

Consequently, the physical demands of EP resulted in many women reporting adverse mental health outcomes. The workload was also associated with mental fatigue and emotional exhaustion, with women stating that the process of EP was ‘too much at times’:

*“I think so many people give up as washing/drying/sterilising 8+ times a day on top of trying to raise a newborn with no sleep absolutely becomes too much at times.”*

(P)

For many women, simply having support people acknowledge the extra workload and dedication of EP mothers was suggested as an important way to validate their experiences:

*“Support the mother, ask what they can do to help. Offer resources that are appropriate. Respect her decision, acknowledge how difficult it is and the effort she’s making to provide breastmilk for her baby.”*

(P)

- Subtheme 7 (Support for Mental Health) explored how recognition of maternal mental health reduced the pressure on EP women. Open communication and providing various options and education meant women felt supported to make decisions that worked best for them:

*“The support I had in the NICU give me lots of options and education in breastfeeding and exclusively pumping with how to increase my supply, they gave me lots of support with my low supply and discussed mixed feeding and if it was to become too stressful for me or if it was affecting my mental health they would supportive if I needed to stop for that reason.”*

(P)

## 4. Discussion

Our study found that EP women have high rates of pregnancy complications, lactation challenges and maternal and infant health conditions, which are all known barriers to direct breastfeeding. The main reasons leading to EP were latching issues and mother wanting to know infant milk intake, as well as infant breast refusal and admission to neonatal unit. Despite these complications, as well as the considerable workload to provide breast milk only by pumping and facing multiple EP-specific challenges, the EP women in this study demonstrated resilience in their feeding method, with many intending to provide breast milk for the same duration as their antenatal breastfeeding intentions. The results of this study also highlight a perceived lack of acceptance and effective professional support, and the need for evidence-based, tailored guidelines to better support EP women.

### 4.1. Breastfeeding Intentions of Exclusively Pumping Women and Early Feeding Practices

During pregnancy, most EP women had strong intentions to breastfeed, with around three quarters intending to exclusively direct breastfeed or combine direct breastfeeding with pumping and EBM feeding, for an average of 12 months (Table 7). Despite the significant challenges faced by EP women, those who had already ceased EP at the time of survey completion reported stopping when their infant was 6.0 [4.0, 9.0] months old (Table 1). This contradicts most previous studies conducted in the USA, China, Hong Kong and Singapore, in which EP was associated with much shorter breast milk feeding duration [18,25], and highlights the perseverance and determination of EP women to feed their infants breast milk. Interestingly, in this study, 25% of women ceased EP due to the infant starting direct breastfeeding and 7% due to having large amounts of frozen milk, speculating that for some EP women, breast milk feeding duration was even longer than 6 months (Table 1).

Unsurprisingly, EP women reported high rates of several lactation challenges, with 100% of the cohort reporting at least one challenge (Table 7). Around three quarters of women had perceived issues with latching and 41% experienced nipple pain from either direct breastfeeding and/or pumping. The lactation challenges experienced by these women are similar to those previously reported [15,18]. Latching issues most commonly led to EP (Table 8), which may be explained by flat, short or large nipples, and/or infant health conditions. Interestingly, only a quarter of women cited nipple or breast pain during breastfeeding as a factor leading to EP, despite 41% of EP mothers experiencing it. This suggests that women are driven by extrinsic and/or intrinsic motivations in their decision to express and feed breast milk [24]; they are prioritising their infant’s needs over their own, with the health benefits of breast milk for infants valued over any pain, discomfort, inconvenience and workload of breastfeeding and EP. Despite the common beliefs that EP is easier than solving breastfeeding challenges [52], only 5% of women were planning EP during pregnancy (Table 3) and only 12% of women selected personal choice as one of the multiple reasons for EP (Table 8), which supports previous indications that very few women actually choose to EP [32].

### 4.2. Birth Mode and Prematurity and/or Neonatal Unit Admission Associations

In this cohort, almost a third of infants were preterm, a value four times higher than in general population (Table 6 and Table 9). Two thirds of women experienced an interventional birth, with 60% birthing by caesarean section, and over 14% having an instrumental assisted vaginal delivery. Caesarean section and assisted vaginal births are associated with reduced mobility and increased pain postpartum, often resulting in poorer mental health outcomes and delayed breastfeeding initiation [12]. Over 40% of this cohorts’ infants were admitted to the neonatal unit (Table 6), more than double that of the general population [43] (Table 9). Infant prematurity and neonatal unit admission often go hand in hand, resulting in separation of the mother and infant, thereby disrupting breastfeeding initiation and early skin-to-skin contact [53,54]. Additionally, preterm infants have undeveloped breathing, sucking and swallowing coordination due to poor muscle development and respiratory difficulties [55,56], which often results in a delayed establishment of breastfeeding.

As expected, in this cohort, we have seen multiple differences between mothers of healthy term infants (HT group) and those who experienced preterm birth and/or infant neonatal unit admission (SP group). Being two thirds primiparous (Table 1), women from the SP group also had higher rates of fertility issues (Table 5). Assisted reproductive technology is shown to negatively impact breastfeeding rates at hospital discharge and at 4 months postpartum [57] and is linked to shorter breastfeeding duration [58], due to the increased complications, such as multiple and preterm birth, low birth weight and caesarean section. Women who have had assisted conception may benefit from health professionals’ help to build their confidence to breastfeed.

In line with having premature birth, during pregnancy, women from the SP group were less likely to access breastfeeding information at all and from online and social media sources (Table 3). Further, with higher rates of pregnancy complications (Table 4) and intervention at birth (Table 6), women from the SP group were twice less likely to attempt direct breastfeeding, likely due to their infants’ prematurity and not being able to latch well (Table 6). As result, during the first week after birth, infants from the SP group were less likely to receive breast milk directly from the breast, and more likely to have EBM and/or donor milk compared with infants from the HT group (Table 6). With less breastfeeding attempts, women from the SP group less frequently reported latching issues and damaged/painful nipples from direct breastfeeding (Table 7). They were also less likely to report both as reasons leading to EP, in addition to infant breast refusal and slow weight gain, which were reported more frequently by HT group participants (Table 8). Interestingly, managing pumping around infant care and discomfort caused by pumping represented less EP challenges to women from the SP group (Table 8), potentially due to some of them not caring for their infants full-time early postpartum.

Expectedly, women from the SP group were more likely to report infant health conditions and having multiple birth infants as reasons leading to EP. The reduced ability of preterm infants to directly breastfeed due to immature suckling skills or the impact of an infant’s health condition [59,60] may have also contributed to these findings. In the SP group, there was a clear reason for EP that would have been fully supported by neonatal unit staff, who understand value of human milk over commercial milk formula. However, for healthy term infants (HT group), there is a predominantly societal expectation that infants will just directly breastfeed if mothers try hard enough. As result, these women are likely to receive less support and more judgement; women likely feel more conflicted about EP than women who have an obvious (infant) reason to EP and support to do so.

Mothers of infants admitted to the NICU face additional stress and pressures associated with having an unwell infant, resulting in adverse mental health outcomes such as anxiety and depression [61,62,63], which are known to further compound breastfeeding challenges [13]. Surprisingly, there was no difference in the self-reported depression and anxiety rates when compared by prematurity/NICU admission status in this cohort. Over half of the survey cohort reported one or more maternal health condition, with particularly high rates of anxiety and depression (Table 9). The higher rates of these self-reported mental health conditions seen in the primiparous group are potentially due to the fact that they have not yet established the social support networks needed on their parenting journey [64,65,66]. Particularly, in this cohort, primiparous EP women were less likely to know of other EP women, so they may have felt more isolated, alone, and judged than multiparous women. However, it is not known if the anxiety or depression were pre-existing conditions, or whether the women developed these mental health conditions during their lactation.

### 4.3. Experience of Care by Health Professionals

We found that despite 81% of women seeking out the expertise of lactation consultants and 53% of midwives, at least two thirds were still unable to resolve their difficulties with direct breastfeeding, reporting infant latch issues (62%) and breast refusal (34%) as main reasons leading to EP (Table 8). Further, our findings indicate that only half of the women perceived the lactation consultants to be supportive or encouraging of EP, with a large proportion either neutral, not understanding/confused or judgmental about EP (Figure 3) or treating it as a temporary solution. Similar responses were reported for community child health nurses and doctors, with approximately 45% perceived as supportive or encouraging. Disturbingly, women reported that health professionals and the public do not perceive providing breast milk via EP as an equivalent to breastfeeding (Section 3.6.1 and Section 3.6.2), making them feel unvalidated. The discontinuity between the internationally accepted WHO definition of breastfeeding as providing breast milk to the infant and the cultural perception of “breast is best” may cause emotional distress for EP women, who are already doing the double load of pumping, cleaning and feeding the infant and are faced with the physical and psychological toll of a non-nursing relationship [52].

Perceived neutral or negative responses to EP may indicate a key gap in health professionals’ attitudes, knowledge and understanding of EP, and/or may reflect the impact of ongoing maternal stress on more negative interpretations of others’ behaviours and intentions [67]. This population is likely particularly vulnerable given their high reported rates of anxiety and depression (Table 5) and ongoing lactation challenges (Table 7). Perceived negative reactions and invalidation from health professionals can compound feelings of inadequacy or mental distress and be associated with reduced self-esteem and depression, further contributing to declining mental health [68]. These findings are complemented by qualitative Theme 1, with many women identifying a key area for improvement in health professionals care as respecting the mother’s EP journey without judgement or shame (Section 3.6.1). Health professionals working with EP women should be mindful of the important role they play in promoting confidence by supporting EP women’s feeding decisions and using positive language and encouragement. Midwives particularly can enhance the care of EP women by maintaining their knowledge and providing specific information about EP and pumping in general, as access to lactation consultants’ services after birth tends to be delayed for some women, with substantial differences in access between public and private settings [12].

Responses relating to health professionals’ confusion likely reflect variable levels of knowledge of EP or pumping in general, highlighting a gap in the education of some health professionals on supporting dyads with latching difficulties or breast refusal, and EP. Theme 2 of the qualitative findings supports this, with many women citing that they felt that lactation consultants and other health professionals were undereducated on pumping, particularly lacking knowledge on flange sizing and how to measure the nipples, and were unable to provide practical EP advice such as tailored pumping schedules and how to maintain milk supply (Section 3.6.2). To date, there has been no published or limited data on various EP characteristics, such as pumping and feeding dynamics, and particularly support strategies [18,69]. Findings from this study, together with existing knowledge on the physiology of milk production, breast pump flange fitting, and milk storage guidelines, can be used to educate lactation consultants and other health professionals on EP so that they are able to guide and support women on their EP journey.

### 4.4. Challenges Associated with Exclusive Pumping

EP women experienced considerable challenges, particularly in relation to time constraints associated with pumping, and managing pumping around the care of their infant (and other children) (Table 7). This was corroborated by the qualitative findings fin Theme 3, where women stated that EP was associated with a significant physical workload that was underappreciated by their support people (Section 3.6.3). This underpins previous work, in which EP women also highlighted the workload associated with pumping, feeding, cleaning and sterilising pumping equipment, as well as milk storage [15,18].

Over 40% of women perceived that they had low milk supply, which is similar to previously reported values of 39% [70] and 44% [49], whilst around a quarter reported an oversupply during their EP lactation experience (Table 7). One third of the survey cohort supplemented their infants with commercial milk formula during the first week postpartum, which was independent of prematurity/neonatal unit admission (Table 6). While this indicates that some women may have been unable to meet their infant’s needs through EBM, this is not different from previously reported frequent physical but non-medically indicated formula use during hospital stay reported for healthy term breastfed infants [12,71]. It is also possible that women have previously had low milk supply that was resolved; nevertheless, low milk supply is a common concern, particularly among primiparous women, which may be true or perceived [49]. Most women reported that their breasts grew by at least one bra cup size during pregnancy (75%) and that they perceived that their breast density changed (81%) (Table 4). The absence of/minimal breast growth in pregnancy has recently been associated with low milk production [72], which is sometimes a cause of breast refusal that may lead to EP [73]. Further research is needed to understand what is considered to be low milk supply in this unique population, who can measure their milk production on a daily basis, and whether this arises from a lack of information.

Half of the women found the cost of pumping supplies to be a challenge, even in this highly educated cohort from high-income settings (Table 8). Returning to paid employment is also often cited as a challenge that breastfeeding women experience [9]; however, only 12% of EP women cited work commitments as a challenge in association with EP. Whilst Australian paid maternity leave is generally shorter than that in many European countries (26 weeks), in this predominantly Australian cohort, women were more likely to return to work later in their postpartum year. To maintain milk supply, 71% of Australian women have been reported to express breast milk at work [74], and pumping skills and routines of EP women are typically already well established. Further, the increased availability of wearable breast pumps may ease the burden of negotiating lactation breaks and private workplace settings in which to pump [75].

### 4.5. Study Strengths and Limitations

The strength of this study lies in its large sample size and a mixed-methods approach, capturing both quantitative and qualitative data to provide an overview of the EP population. However, this study has some limitations. Whilst we aimed at a large sample and clearly defined the selection criteria for participants, study participants were recruited online and respondents with biases may select themselves into the sample. People with strong opinions are more likely to respond and we could have an over-representation of those who felt unsupported or lacked adequate information and education from health professionals, and those who were well supported may not bother to respond to a survey. Most women who completed the survey were highly educated, which could affect the results as these women may have a better understanding of the value of breastfeeding and breast milk [76]. As such, results from this study may not generalise to broader populations and not be representative of the experiences of EP women with lower education levels or from low-income settings, particularly. Higher education is positively associated with socioeconomic status, so it is likely that EP women who completed the survey are of higher socioeconomic status; however, we did not formally assess this. The characterisation of our respondents also suggests we have not captured culturally and linguistically diverse or disadvantaged populations, which may bias the results. While the survey was available via social media sites in several countries, the study cohort were predominantly residing in Australia. Therefore, findings may also not reflect those of EP women in other high-income countries (including France, New Zealand, UK and USA). As structural and societal supports differ between countries, with shorter and unpaid maternity leave in some countries resulting in earlier return to paid employment and reliance on pumping, replicating this study in other countries may lead to a better understanding of the unique needs of EP women in different settings, enabling tailored guidance.

## 5. Conclusions

In this study of EP women, we found a high prevalence of pregnancy complications and infant health conditions, potentially making them a high-risk population for early direct breastfeeding cessation. The main reasons leading to EP were latching issues and parents wanting to know infant milk intake, as well as infant breast refusal and admission to neonatal unit. However, despite these challenges, EP women demonstrated resilience in their journey to provide their infant breast milk. Most EP women sought professional support when direct breastfeeding challenges arose, and persisted with EP when the issues were not resolved. Women reported judgement and a lack of professional support, as well as inadequate practical pumping advice or emotional care to support them through the physical and mental workload involved in this feeding method. This study has highlighted the need for better education about pumping and EP for health professionals working with breastfeeding women. Further research will provide information that can be used by healthcare professionals to inform their clinical practice and provide better guidance and support for EP women.

## Figures and Tables

**Table 1:** Exclusive pumping status, infant age at cessation and reasons for cessation of exclusive pumping.

Characteristics	Total *n* = 195	HT Group *n* = 105	SP Group *n* = 90	*p*-Value ^2^
Infant age at survey (months)	7.1 [3.9, 14.0] ^1^	6.3 [3.4, 11.9]	8.1 [3.9, 16.6]	0.061
Primiparous	120 (61.5)	60 (57.1)	60 (66.7)	**0.037**
EP status				
Currently EP	113 (58.0)	61 (58.1)	52 (57.8)	0.96
Previously EP	82 (42.0)	44 (41.9)	38 (42.2)	0.96
EP ceased ^3^	*n* = 81	*n* = 44	*n* = 38	
Infant age at EP cessation (months) ^3^	6.0 [4.0, 9.0]	6.0 [4.0, 9.0]	5.0 [3.0, 10.5]	0.67
Reasons for EP cessation ^4^				
Too difficult to pump and take care of infant	34 (42.0)	19 (44.2)	15 (39.5)	0.67
Low milk supply	22 (27.2)	10 (23.3)	12 (31.6)	0.40
Infant started to direct BF	20 (24.7)	10 (23.3)	10 (26.3)	0.75
Pumping was too painful or uncomfortable	6 (7.4)	5 (11.6)	1 (2.6)	0.21
Having large amounts of frozen milk	6 (7.4)	5 (11.6)	1 (2.6)	0.21
Mental health	5 (6.2)	4 (9.3)	1 (2.6)	0.36
Other ^5^	24 (29.6)	13 (30.2)	11 (28.9)	0.90

^1^ Data are *n* (%) or median [Q1, Q3]. ^2^ *p*-value indicates significant difference between HT (healthy term group—women with term infants that did not have a neonatal unit admission) and SP (sick/preterm group—women with infants that were born preterm (<37 weeks’ birth gestation) and/or had a neonatal unit admission) groups using unpaired Student’s *t*-test, Chi-square or Fisher’s exact test where appropriate; bold font indicates a significant difference. ^3^ Those that ceased EP prior to survey participation; ^4^ sum of percentages > 100%, as participants could select more than one response. ^5^ Other included reasons such as mastitis, infants having solids, trying to conceive again, reaching breastfeeding goals, infant allergies, returning to work and not wanting to EP anymore. BF, breastfeeding; EP, exclusive pumping.

**Table 2:** Exclusively pumping women’s demographics.

Characteristics	Total *n* = 195	HT Group *n* = 105	SP Group *n* = 90	*p*-Value ^2^
Country				
Australia	180 (92.3) ^1^	93 (88.6)	87 (96.7)	**0.034**
United States of America	8 (4.1)	6 (5.7)	2 (2.2)	0.29
United Kingdom	3 (1.5)	2 (1.9)	1 (1.1)	1.00
New Zealand	3 (1.5)	3 (2.9)	0 (0.0)	0.18
France	1 (0.5)	1 (0.9)	0 (0.0)	1.00
Education level				
High school	22 (11.3)	13 (12.4)	9 (10.0)	0.66
Certificate/diploma	45 (23.1)	24 (22.9)	21 (23.3)	0.94
Bachelor’s degree or above	128 (65.6)	68 (64.8)	60 (66.7)	0.78
Marital status				
Never married or de facto	10 (5.1)	7 (6.7)	3 (3.3)	0.35
Married or de facto	182 (93.3)	98 (93.3)	84 (93.3)	1.00
Separated or divorced	3 (1.6)	0 (0.0)	3 (3.3)	0.097
Pre-pregnancy BMI (kg/m^2^)	27.6 [23.8, 32.5]	28.7 [25.1, 33.1]	26.7 [22.5, 31.0]	**0.001**
Current BMI (kg/m^2^)	*n* = 192	*n* = 105	*n* = 87	
	29.1 [24.8, 32.8]	30.1 [26.3, 33.9]	27.5 [23.3, 32.1]	**0.001**

^1^ Data are n (%) or median [Q1, Q3]. ^2^ *p*-value indicates significant difference between HT (healthy term group—women with term infants that did not have a neonatal unit admission) and SP (sick/preterm group—women with infants that were born preterm (<37 weeks’ birth gestation) and/or had a neonatal unit admission) groups using unpaired Student’s *t*-test, Chi-square or Fisher’s exact test where appropriate; bold font indicates a significant difference. BMI, body mass index.

**Table 3:** Breastfeeding intentions and information seeking of exclusively pumping women during pregnancy.

Characteristics	Total *n* = 195	HT Group *n* = 105	SP Group *n* = 90	*p*-Value ^2^
Planned infant feeding method				
Exclusive BF	97 (49.7) ^1^	58 (55.2)	39 (43.3)	0.097
Mix of breastfeeding and pumping	51 (26.2)	28 (26.7)	23 (25.6)	0.86
Mix of BF, pumping and formula feeding	15 (7.7)	5 (4.8)	10 (11.1)	0.11
Exclusive bottle feeding of EBM	10 (5.1)	4 (3.8)	6 (6.7)	0.52
Exclusive formula feeding	4 (2.1)	0 (0.0)	4 (4.4)	**0.044**
No plans	18 (9.2)	10 (9.5)	8 (8.9)	1.00
Intended BF length (months)	*n* = 192	*n* = 103	*n* = 89	
	12.0 [9.8, 14.5]	12.0 [12.0, 12.0]	12.0 [8.0, 18.0]	0.94
BF information sources accessed ^3^	*n* = 195	*n* = 105	*n* = 90	
Did not access BF information	43 (22.1)	11 (10.5)	32 (35.6)	**<0.001**
Midwife/OBGYN nurse	91 (46.7)	55 (52.4)	36 (40.0)	0.084
Online/social media	84 (43.1)	58 (55.2)	26 (28.9)	**<0.001**
Lactation consultant	65 (33.3)	38 (36.2)	27 (30.0)	0.36
BF class	48 (24.6)	24 (22.9)	24 (26.7)	0.54
Online forums/groups	42 (21.5)	27 (25.7)	15 (16.7)	0.13
Obstetrician	28 (14.4)	17 (15.2)	12 (13.3)	0.61
GP/family physician	22 (11.3)	13 (12.4)	9 (10.0)	0.66
Doula	2 (1.0)	2 (1.9)	0 (0.0)	0.50
Other ^4^	10 (5.1)	8 (7.6)	2 (2.2)	0.11

^1^ Data are n (%) or median [Q1, Q3]. ^2^ *p*-value indicates significant difference between HT (healthy term group—women with term infants that did not have a neonatal unit admission) and SP (sick/preterm group—women with infants that were born preterm (<37 weeks’ birth gestation) and/or had a neonatal unit admission) groups using unpaired Student’s *t*-test, Chi-square or Fisher’s exact test where appropriate; bold font indicates a significant difference. ^3^ Sum of percentages > 100%, as participants could select more than one response. ^4^ Other included sources such as online/live classes, family members, Australian Breastfeeding Association, podcasts and books. BF, breastfeeding; EBM, expressed breast milk; GP, general practitioner; OBGYN, obstetrics and gynaecology.

**Table 4:** Pregnancy details of exclusively pumping women.

Characteristics	Total *n* = 195	HT Group *n* = 105	SP Group *n* = 90	*p*-Value ^2^
Number of births for this pregnancy				
Singleton birth	181 (92.8) ^1^	104 (99.0)	77 (85.6)	**<0.001**
Multiple births (twins)	14 (7.2)	1 (1.0)	13 (14.4)	**<0.001**
Pregnancy complications ^3^				
No complications	68 (34.9)	50 (47.6)	18 (20.0)	**<0.001**
Gestational diabetes mellitus	44 (22.6)	22 (21.0)	22 (24.4)	0.56
Hypertension	34 (17.4)	17 (16.2)	17 (18.9)	0.62
Foetal growth restriction	34 (17.4)	6 (5.7)	28 (31.1)	**<0.001**
Preeclampsia	29 (14.9)	6 (5.7)	23 (25.6)	**<0.001**
Placental insufficiency	16 (8.2)	2 (1.9)	14 (15.6)	**<0.001**
Anaemia	14 (7.2)	10 (9.5)	4 (4.4)	0.27
Other ^4^	42 (21.5)	13 (12.4)	29 (32.2)	**<0.001**
Breast growth during pregnancy				
Breasts grew by one bra cup size or more	147 (75.4)	74 (70.5)	73 (81.1)	0.086
No breast growth	33 (16.9)	19 (18.1)	14 (15.6)	0.64
Unsure	15 (7.7)	12 (11.4)	3 (3.3)	0.056
Perceived change in breast density				
Breast density increased	157 (80.5)	81 (77.1)	76 (84.4)	0.20
Breast density did not change	29 (14.9)	18 (17.1)	11 (12.2)	0.34
Unsure	9 (4.6)	6 (5.7)	3 (3.3)	0.51

^1^ Data are *n* (%). ^2^ *p*-value indicates significant difference between HT (healthy term group—women with term infants that did not have a neonatal unit admission) and SP (sick/preterm group—women with infants that were born preterm (<37 weeks’ birth gestation) and/or had a neonatal unit admission) groups using Chi-square or Fisher’s exact test where appropriate; bold font indicates a significant difference. ^3^ Sum of percentages > 100%, as participants could select more than one response. ^4^ Other included pregnancy complications such as placenta previa, incompetent cervix and various foetal health conditions.

**Table 5:** Exclusively pumping women’s health and breast conditions.

Characteristics	Total *n* = 195	HT Group *n* = 105	SP Group *n* = 90	*p*-Value ^2^
Maternal health conditions ^3^				
No health conditions	84 (43.1) ^1^	49 (46.7)	35 (38.9)	0.27
Anxiety ^4^	62 (31.8)	31 (29.5)	31 (34.4)	0.46
Depression ^4^	39 (20.0)	21 (20.0)	18 (20.0)	1.00
Fertility issues	31 (15.9)	10 (9.5)	21 (23.3)	**0.009**
Polycystic ovary syndrome	29 (14.9)	16 (15.2)	13 (14.4)	0.88
Thyroid disorder	12 (6.2)	6 (5.7)	6 (6.7)	0.75
Insulin resistance	8 (4.1)	4 (3.8)	4 (4.4)	1.00
Diabetes	1 (0.5)	0 (0.0)	1 (1.1)	0.46
Other	25 (12.8)	14 (13.3)	11 (12.2)	0.82
Breast conditions/surgery ^3^				
No conditions	114 (58.5)	64 (61.0)	50 (55.6)	0.45
Mastitis	35 (18.0)	16 (15.2)	19 (21.1)	0.29
Large nipples	28 (14.4)	19 (18.1)	9 (10.0)	0.15
Nipple piercing	19 (9.7)	11 (10.5)	8 (8.9)	0.81
Flat/short nipples	15 (7.7)	8 (7.6)	7 (7.8)	1.00
Cysts	9 (4.6)	7 (6.7)	2 (2.2)	0.18
Benign lump	3 (1.5)	1 (1.0)	2 (2.2)	0.60
Breast augmentation	2 (1.0)	1 (1.0)	1 (1.1)	1.00
Breast reduction	2 (1.0)	1 (1.0)	1 (1.1)	1.00
Breast lumpectomy	2 (1.0)	1 (1.0)	1 (1.1)	1.00
Breast abscess	1 (0.5)	0 (0.0)	1 (1.1)	0.46
Other	5 (2.6)	4 (3.8)	1 (1.1)	0.38

^1^ Data are *n* (%). ^2^ *p*-value indicates significant difference between HT (healthy term group—women with term infants that did not have a neonatal unit admission) and SP (sick/preterm group—women with infants that were born preterm (<37 weeks’ birth gestation) and/or had a neonatal unit admission) groups using Chi-square or Fisher’s exact test where appropriate; bold font indicates a significant difference. ^3^ Sum of percentages > 100%, as participants could select more than one response. ^4^ Self-reported by participants and may not be clinically diagnosed.

**Table 6:** Infant birth and feeding in the first postpartum week.

Characteristics	Total *n* = 195	HT Group *n* = 105	SP Group *n* = 90	*p*-Value ^2^
Type of birth				
Unassisted vaginal birth	63 (32.3) ^1^	51 (48.6)	12 (13.3)	**<0.001**
Vacuum-assisted vaginal birth	7 (3.6)	6 (5.7)	1 (1.1)	0.13
Forceps-assisted vaginal birth	7 (3.6)	4 (3.8)	3 (3.3)	1.00
Planned caesarean section	46 (23.6)	25 (23.8)	21 (23.3)	0.94
Unplanned caesarean section	72 (36.9)	19 (18.1)	53 (58.9)	**<0.001**
Infant characteristics				
Birth gestation (weeks)	38.6 [35.3, 39.4]	39.1 [38.7, 40.1]	34.6 [30.1, 37.6]	**<0.001**
Preterm infants (all; <37 weeks)	62 (31.8)	0 (0.0)	62 (68.9)	**<0.001**
Extremely preterm (<28 weeks)	18 (9.2)	0 (0.0)	18 (20.0)	**<0.001**
Very preterm (28–32 weeks)	14 (7.2)	0 (0.0)	14 (15.6)	**<0.001**
Moderate-to-late preterm (32–37 weeks)	30 (15.4)	0 (0.0)	30 (33.3)	**<0.001**
Term (>37 weeks)	133 (68.2)	105 (100.0)	28 (31.1)	**<0.001**
Neonatal unit admission	83 (42.6)	0 (0.0)	83 (92.2)	**<0.001**
Infant health condition	52 (26.7)	12 (11.4)	40 (44.4)	**<0.001**
Direct BF attempted	152 (78.0)	102 (97.1)	50 (55.6)	**<0.001**
Infant latch in 1st week	*n* = 152	*n* = 102	*n* = 50	
Infant did not latch well most/all of the time	71 (46.7)	40 (39.2)	31 (62.0)	**0.008**
Latch caused pain	50 (32.9)	38 (37.3)	12 (24.0)	0.10
Infant latched well most/all of the time	31 (20.4)	24 (23.5)	7 (14.0)	0.20
Infant diet in the 1st week ^3^	*n* = 195	*n* = 105	*n* = 90	
EBM	165 (84.6)	81 (77.1)	84 (93.3)	**0.002**
Breast milk directly from the breast	89 (45.6)	70 (66.7)	19 (21.1)	**<0.001**
Commercial milk formula	64 (32.8)	38 (36.2)	26 (28.9)	0.28
Donor human milk	18 (9.2)	2 (1.9)	16 (17.8)	**<0.001**
Other	4 (2.1)	1 (1.0)	3 (3.3)	0.34
Duration of attempting direct BF	*n* = 152	*n* = 102	*n* = 50	
1st week	30 (19.7)	22 (21.6)	8 (16.0)	0.52
1st month	43 (28.3)	31 (30.4)	12 (24.0)	0.41
2–3 months	38 (25.0)	23 (22.5)	15 (30.0)	0.32
3–6 months	14 (9.2)	12 (11.8)	2 (4.0)	0.15
Direct BF still attempted	27 (17.8)	14 (13.7)	13 (26.0)	0.15

^1^ Data are n (%) or median [Q1, Q3]. ^2^ *p*-value indicates significant difference between HT (healthy term group—women with term infants that did not have a neonatal unit admission) and SP (sick/preterm group—women with infants that were born preterm (<37 weeks’ birth gestation) and/or had a neonatal unit admission) groups using unpaired Student’s *t*-test, Chi-square or Fisher’s exact test where appropriate; bold font indicates a significant difference. ^3^ Sum of percentages > 100%, as participants could select more than one response. BF, breastfeeding; EBM, expressed breast milk.

**Table 7:** Lactation challenges experienced and breastfeeding services/information sources accessed by exclusively pumping women.

Characteristics	Total *n* = 195	HT Group *n* = 105	SP Group *n* = 90	*p*-Value ^2^
Lactation challenges ^3^				
No challenges	0 (0.0) ^1^	0 (0.0)	0 (0.0)	1.00
Latching issues	144 (73.9)	85 (81.0)	59 (65.6)	**0.015**
Perceived low milk supply	84 (43.1)	42 (40.0)	42 (46.7)	0.35
Blocked ducts	83 (42.6)	46 (43.8)	37 (41.1)	0.70
Damaged/painful nipples from BF	80 (41.0)	57 (54.3)	23 (25.6)	**<0.001**
Damaged/painful nipples from pumping	80 (41.0)	37 (35.2)	43 (47.8)	0.076
Oversupply	58 (29.7)	28 (26.7)	30 (33.3)	0.31
Mastitis	50 (25.6)	26 (24.8)	24 (26.7)	0.76
Nipple bleb	44 (22.6)	28 (26.7)	16 (17.8)	0.11
BF services/information sources accessed ^3^				
No BF services or information accessed	9 (4.6)	4 (3.8)	5 (5.6)	0.74
Lactation consultant	157 (80.5)	84 (80.0)	73 (81.1)	0.85
Midwife	104 (53.3)	56 (53.3)	48 (53.3)	1.00
Websites	88 (45.1)	53 (50.5)	35 (38.9)	0.11
Community child health nurse	74 (37.9)	45 (42.9)	29 (32.2)	0.13
General practitioner	48 (24.6)	25 (23.8)	23 (25.6)	0.78
Breastfeeding helpline	44 (22.6)	25 (23.8)	19 (21.1)	0.65
Mothers group	41 (21.0)	29 (27.6)	12 (13.3)	**0.015**
Paediatrician	29 (14.9)	19 (18.1)	10 (11.1)	0.098
Obstetrician	21 (10.8)	9 (8.6)	12 (13.3)	0.36
Other	12 (6.2)	8 (7.6)	4 (4.4)	0.39

^1^ Data are *n* (%). ^2^ *p*-value indicates significant difference between HT (healthy term group—women with term infants that did not have a neonatal unit admission) and SP (sick/preterm group—women with infants that were born preterm (<37 weeks’ birth gestation) and/or had a neonatal unit admission) groups using Chi-square or Fisher’s exact test where appropriate; bold font indicates a significant difference. ^3^ Sum of percentages > 100%, as participants could select more than one response. BF, breastfeeding.

**Table 8:** Factors leading to exclusive pumping and exclusive pumping challenges.

Characteristics	Total *n* = 195	HT Group *n* = 105	SP Group *n* = 90	*p*-Value ^2^
When did the woman first learn about EP?				
Before this pregnancy	83 (42.6) ^1^	52 (49.5)	31 (34.4)	**0.034**
During this pregnancy	26 (13.3)	14 (13.3)	12 (13.3)	1.00
After the birth of this infant	79 (40.5)	36 (34.3)	43 (47.8)	0.056
Unsure	7 (3.6)	3 (2.9)	4 (4.4)	0.71
Factors leading to EP ^3^				
Latching issues	121 (62.1)	78 (74.3)	43 (47.8)	**<0.001**
Mother wanted to know milk intake	69 (35.4)	41 (39.0)	28 (31.1)	0.25
Infant breast refusal	66 (33.9)	44 (41.9)	22 (24.4)	**0.010**
Neonatal unit admission	64 (32.8)	0 (0.0)	64 (71.1)	**<0.001**
Sharing infant feeding responsibility	56 (28.7)	31 (29.5)	25 (27.8)	0.79
Pain during direct breastfeeding	49 (25.1)	38 (36.2)	11 (12.2)	**<0.001**
Infant health condition	42 (21.5)	16 (15.2)	26 (28.9)	**0.021**
Personal choice	24 (12.3)	13 (12.3)	11 (12.2)	0.97
Low milk supply	22 (11.3)	12 (11.4)	10 (11.1)	0.94
Returning to paid work	17 (8.7)	13 (12.3)	4 (4.4)	0.073
Slow infant weight gain	11 (5.6)	10 (9.5)	1 (1.1)	**0.012**
Multiple infants	10 (5.1)	1 (1.0)	9 (10.0)	**0.006**
Other ^4^	28 (14.4)	16 (15.2)	11 (12.2)	0.54
EP challenges experienced ^3^				
Time constraints of pumping	166 (85.1)	91 (86.7)	75 (83.3)	0.51
Managing pumping around infant care	162 (83.1)	94 (89.5)	68 (75.6)	**0.010**
Nighttime pumping	141 (72.3)	80 (76.2)	61 (67.8)	0.19
Discomfort caused by pumping	111 (56.9)	68 (64.8)	43 (47.8)	**0.017**
Sore nipples	99 (50.8)	49 (46.7)	50 (55.6)	0.22
Cost of pumping supplies	98 (50.3)	54 (51.4)	44 (48.9)	0.72
Painful breasts	84 (43.1)	45 (42.9)	39 (43.3)	0.95
Low milk supply	81 (41.5)	38 (36.2)	43 (47.8)	0.10
EBM storage	74 (38.0)	41 (39.0)	33 (36.7)	0.73
Judgement	73 (37.4)	43 (41.0)	30 (33.3)	0.27
Lack of knowledge	63 (32.3)	35 (33.3)	28 (31.1)	0.74
Oversupply	52 (26.7)	28 (26.7)	24 (26.7)	1.00
Thawing of frozen EBM	42 (21.5)	23 (21.9)	19 (21.1)	0.89
Faulty pump	27 (13.9)	14 (13.3)	11 (12.2)	0.82
Work commitments	23 (11.8)	12 (10.0)	9 (10.0)	0.82
Other	16 (8.2)	9 (8.6)	7 (7.8)	1.00

^1^ Data are *n* (%). ^2^ *p*-value indicates significant difference between HT (healthy term group—women with term infants that did not have a neonatal unit admission) and SP (sick/preterm group—women with infants that were born preterm (<37 weeks’ birth gestation) and/or had a neonatal unit admission) groups using Chi-square or Fisher’s exact test where appropriate; bold font indicates a significant difference. ^3^ Sum of percentages > 100%, as participants could select more than one response. ^4^ Other included factors leading to EP such as difficulty with feeding twins, inadequate milk transfer, oversupply and fast letdown and sensory issues preventing them from trying to latch the infant. EBM, expressed breast milk; EP, exclusive pumping.

**Table 9:** Maternal and infant health and breastfeeding conditions of exclusively pumping Australian women compared with Australian general population data.

Characteristics	EP Women Frequency *n* = 180	General Population Frequency	Total Sample Size	*p*-Value ^2^
Mode of birth				
Caesarean section	112 (62.2) ^1^	114,440 (39.0) ^3^	293,615	**<0.001**
Pregnancy complications and infant health conditions				
GDM	42 (23.3)	53,900 (18.0) ^4^	299,624	0.063
Gestational hypertension	31 (17.2)	8758 (3.2) ^5^	267,654	**<0.001**
Preterm birth	58 (32.2)	23,888 (8.4) ^6^	285,485	**<0.001**
Neonatal unit admission	81 (45.0)	29,548 (17.9) ^6^	164,823	**<0.001**
Maternal health conditions				
Anxiety/depression	91 (50.5)	1,917,557 (15.7) ^7^	12,213,920	**<0.001**
Polycystic ovary syndrome	27 (15.0)	31 (12.2) ^8^	434	0.40
Lactation challenges				
Nipple pain	141 (78.3)	169 (36.0) ^9^	649	**<0.001**
Percieved low milk supply	79 (43.9)	171 (44.2) ^10^	567	0.95
Mastitis	47 (26.1)	206 (17.0) ^11^	1373	**0.** **004**

^1^ Data are *n* (%). ^2^ *p*-value indicates the difference between EP and general population groups using Chi-square test; bold font indicates a significant difference. ^3^ *n* = 293,435, 2022 [43]; ^4^ *n* = 299,444, 2022 [44]; ^5^ *n* = 267,474, 2022 [43]; ^6^ *n* = 285,305, 2023, preterm births; *n* = 164,643, 2023, admission to a special care nursery or NICU [43]; ^7^ *n* = 12,213,740, 2017–2018, females [46]; ^8^ *n* = 254, 2012–2016 [45]; ^9^ *n* = 469, 2011, nipple pain as a reason for consultation at the breastfeeding centre [47]; ^10^ *n* = 387, 2021 [49]; ^11^ *n* = 1193, 1999–2001 [48]. EP, exclusive pumping; GDM, gestational diabetes mellitus.

## Data Availability

The data presented in this study are available on request from the corresponding author due to ethical restrictions.

## Supplementary Materials

The following supporting information can be downloaded at: https://www.mdpi.com/article/10.3390/healthcare14101361/s1, Supplementary Materials S1: Survey questions.

## Abbreviations

The following abbreviations are used in this manuscript:

BF	Breastfeeding
BMI	Body mass index
EBM	Expressed breast milk
EP	Exclusive/exclusively pumping
GDM	Gestational diabetes mellitus
HT	Healthy term
NICU	Neonatal intensive care unit
REDCap	Research Electronic Data Capture
SP	Sick/preterm
WHO	World Health Organization
