# Supplementary material for: Maternal Experiences with Exclusive Pumping—An Online Survey
Source: Healthcare (Basel). 2026 May 15;14(10):1361. doi: 10.3390/healthcare14101361 (PMC13205383; doi:10.3390/healthcare14101361)
Supplement: Supplementary file 1 [file healthcare-14-01361-s001.zip › healthcare-4261132-supplementary.pdf]

healthcare

MDPI

Article

Maternal Experiences with Exclusive Pumping–an Online Survey

Zoya Gridneva [1,2,3,*], Jacki L. McEachran [1,2,3], Demelza J. Ireland [4], Sharon L. Perrella [1,2,3] and Donna T. Geddes [1,2,3]

[1] School of Molecular Sciences, The University of Western Australia, Crawley, WA 6009, Australia; jacki.mceachran@uwa.edu.au (J.L.M.); sharon.perrella@uwa.edu.au (S.L.P.); donna.geddes@uwa.edu.au (D.T.G.)
[2] ABREAST Network, Perth, WA 6000, Australia
[3] UWA Centre for Human Lactation Research and Translation, Crawley, WA 6009, Australia
[4] School of Biomedical Sciences, The University of Western Australia, Crawley, WA 6009, Australia; demelza.ireland@uwa.edu.au (D.J.I.)
* Correspondence: zoya.gridneva@uwa.edu.au (Z.G.); Tel.: +61-8-6488-4467

Supplementary Material S1: Survey Questions

Characteristics and pumping dynamics of women that exclusively pump for their infants

Survey Questions:

For this study exclusive pumping (EP) is defined as only feeding the baby pumped/expressed breast milk. That is, the baby does not drink breast milk directly from the breast. A mother is still considered to exclusively pump if the baby also fed formula. Please note that the term ‘pumping’ refers to any form of breast milk expression including hand expression, manual or electric pumping. You are welcome to answer the survey questions if you are currently exclusively pumping or have exclusively pumped in the past and your baby is under 24 months of age.

1. Are you currently or have you previously exclusively pumped for your baby that is currently under the age of 24 months?

- o No (end of survey)
- o Yes, I am currently exclusively pumping (Skip Q2 and Q3, go to Q4)
- o I have stopped exclusively pumping and my baby is under the age of 24 months (if yes, drop-down Q2 and Q3):

2. How old was your baby when you stopped exclusively pumping?

_________ months

3. What were your reasons for stopping exclusive pumping? (Check all that apply)

- o Baby started to feed from the breast
- o It became too difficult to pump and feed/take care of my baby
- o I had low milk supply
- o Pumping was too painful or uncomfortable
- o Other: ________

Background information

4. **What is your date of birth**? dd/mm/yyyy

5. **What country do you currently live in? ________**

6. **What is the highest level of education you have completed?**
- o Primary school
- o High school
- o Certificate or diploma
- o Bachelor degree or above

7. **What is your marital status?**
- o Never married or de facto
- o Married or de facto
- o Separated or divorced
- o Widowed

8. **What is your height? (Please state whether in cm or feet and inches)**
____

9. **What was your pre pregnancy weight? (Please state whether in kg or lbs)**
____

10. **What is your current weight? (Please state whether in kg or lbs) __________**

11. **Do you have any health conditions? (Check all that apply)**
- o Anxiety
- o Depression
- o Diabetes (diagnosed before this pregnancy)
- o Fertility issues requiring assisted reproduction for this pregnancy
- o Thyroid disorder
- o Insulin resistance
- o Pituitary disease
- o Polycystic ovary syndrome
- o No health conditions
- o Other (please list): _____

12. **Have you had any of the following nipple or breast conditions or surgery? (Check all that apply)?**
- o No previous surgery or piercing
- o Nipple piercing
- o Nipple surgery
- o Breast augmentation (enlargement)
- o Breast reduction
- o Breast lumpectomy
- o Inverted nipples
- o Flat/short nipples
- o Large nipples
- o Cysts
- o Benign lumps (e.g. Fibroadenoma)

- o Breast abscess
- o Mastitis
- o Other (please list): _____

13. Are you a first-time mother?
- o Yes (If yes, skip Q14)
- o No

14. Did you exclusively pump for your previous baby / babies?
- o Yes
- o No (If no go to Q13)

Pregnancy

15. Did your breasts grow by one bra cup size or more during pregnancy?
- o Yes
- o No
- o Unsure

16. Did your breasts become more heavy or 'dense' during pregnancy?
- o Yes
- o No
- o Unsure

17. Was this a multiple birth pregnancy?
- o Yes
- o No (If no, skip Q18, go to Q19)

18. How many babies? (If yes go to Q17)
- o 2
- o 3
- o 4+

19. During pregnancy, what was your planned method of feeding your baby?
- o Exclusive breastfeeding
- o Exclusive bottle feeding of my pumped milk
- o Exclusive formula feeding (if selected skip Q20, go to Q21)
- o Both breastfeeding and pumping
- o Breastfeeding and pumping and formula feeding
- o I didn't have any set feeding plans during pregnancy

20. During pregnancy, how long did you intend to breastfeed / provide breast milk for your baby? _______ months

21. Did you have any pregnancy complications? (Check all that apply)
- o No complications
- o High blood pressure
- o Gestational diabetes
- o Anaemia
- o Pre-eclampsia or eclampsia
- o Fetal growth restriction

- o Placental insufficiency
- o Other (please list): _____

22. What breastfeeding information sources did you access during pregnancy? (Check all that apply)

- o I did not access any breastfeeding information during pregnancy
- o Midwife / OBGYN nurse
- o Lactation consultant / IBCLC
- o General practitioner / family physician
- o Obstetrician / OBGYN
- o Breastfeeding class
- o Doula
- o Online / social media
- o Online forums / groups
- o Other (please list): _____

Birth and baby details

23. What is your baby's date of birth? dd/mm/yyyy

24. What was your baby's due date? dd-mm-yyyy

25. What type of birth did you have?

- o Unassisted vaginal birth
- o Vacuum assisted vaginal birth
- o Forceps assisted vaginal birth
- o Planned caesarean birth
- o Unplanned / emergency caesarean birth

26. Was your baby admitted to the neonatal nursery / neonatal intensive care unit (NICU)?

- o Yes
- o No

27. Does your baby have any health conditions?

- o Yes (please list): _______
- o No

Lactation History

28. Did you attempt to breastfeed before starting to exclusively pump?

- o Yes
- o No (if No skip Q's 29 and 30, go to Q31)

29. For how long did you try to feed your baby at the breast?

- o I stopped trying in the first week
- o I stopped trying after the first month
- o I stopped trying after 2-3 months
- o I stopped trying after 3-6 months
- o I still try to breastfeed my baby

30. Please describe your baby's latching in the first week after birth:
- o Baby latched and breastfed well most or all the time
- o Baby's latch caused nipple pain and/or trauma for most or all feeds
- o Baby did not latch to the breast for most or all feeds

31. What did you feed your baby in the first week after birth? (Check all that apply)
- o My breast milk by breastfeeding
- o My expressed breast milk
- o Donor breast milk
- o Commercial milk formula
- o Other: ________

32. Have you experienced any of the following lactation challenges? (Check all that apply)
- o Attachment / latching difficulties
- o Low milk supply
- o Oversupply
- o Damaged / sore nipples related to breastfeeding
- o Damaged / sore nipples related to pumping
- o Mastitis
- o Blocked ducts
- o Nipple bleb / white spot

33. What services / information sources did you access to get help with trying to breastfeed? (Check all that apply)
- o I did not access any breastfeeding services or information for help
- o Community nurse
- o Midwife / OBGYN nurse
- o Lactation consultant / IBCLC
- o General practitioner/ family physician
- o Obstetrician / OBGYN
- o Pediatrician
- o Mothers' groups
- o Websites / social media
- o Breastfeeding hotline (e.g. La Leche League, Australian Breastfeeding Association)
- o Other (please list): _____

34. How helpful were these sources in relation to breastfeeding?

Source	Very helpful	Helpful	Neutral	Unhelpful	Very unhelpful	Not applicable
Community nurse						
Midwife / OBGYN nurse						

Lactation consultant						
General practitioner/family physician						
Obstetrician/OBGYN						
Pediatrician						
Mother's groups						
Websites /social media						
Other						

Pumping

35. When did you first find out that exclusive pumping was an option for feeding babies breast milk?

o Prior to becoming pregnant
o During pregnancy
o After the birth of this baby
o Unsure

36. How would you describe the responses you received from the following support persons about exclusive pumping?

Person	Support/ Encouragement	Neutral	Confusion/ Did not understand	Judgement/ Shame	Not applicable
Self					
Partner					
Extended family					
Friends/ Colleagues					
Visiting midwife					
Community child health nurse					
Lactation consultant					
Doctor/ Physician					

37. Please describe how you think support people and health professionals could better support exclusively pumping mothers? (Insert open text box, no word limit) _____________

38. What factors led you to exclusively pump your milk? (Check all that apply)

- o Latching issues e.g. difficult or painful latch
- o Baby was refusing the breast
- o Nipple / breast pain during breastfeeding
- o Baby in the neonatal nursery / Neonatal Intensive Care Unit
- o I wanted to know how much milk the baby was drinking
- o Baby had a health condition that affected sucking
- o I felt this would be easier with feeding multiple babies (twins, triplets, etc.)
- o I felt this was easier for managing low milk supply
- o I felt this would be easier with returning to paid work
- o Personal choice (preferred pumping to breastfeeding)
- o I wanted to share the feeding responsibilities with my support people
- o Other (please state): ______

39. What challenges have you experienced with exclusive pumping? (Check all that apply)

- o Low milk supply
- o Painful breasts (mastitis, blocked ducts)
- o Sore or damaged nipples
- o Milk oversupply
- o Storage of pumped milk
- o Thawing and heating of frozen breast milk
- o Finding time to pump
- o Judgement from other people
- o Being uncomfortable pumping around other people
- o Work commitments (difficulty finding time or space to pump at work)
- o Managing pumping around baby's feeding and care needs
- o Needing to pump at night when baby is sleeping
- o Lack of knowledge around pumping
- o Cost of pumping supplies
- o Faulty / broken / poor quality pump
- o Other (please state): ____

Questions about your pump/s and pumping methods

40. How do / did you express your milk? (Check all that apply)

- o Hospital grade electric breast pump
- o Electric personal use breast pump
- o Manual pump
- o Wearable pump
- o Hand expression (If hand expression only, skip Q40-52, go to Q53)

41. Do you / did you use more than one pump?

- o Yes
- o No

42. What sort of pump do / did you use the most?

- o Hospital grade electric
- o Personal use electric
- o Manual
- o Wearable
- o Other (please state): ______

43. What is the brand of the breast pump/s you are / were using? _________

44. What is / was the size / diameter of your pump breast shield in millimeters (mm)?

- o ______mm
- o I don't know

45. If using an electric pump, what vacuum / suction settings do / did you use?

- o Low
- o Medium
- o High
- o Other _____
- o Not applicable – I can't change it
- o I don't know

46. Do / did you use a pumping bra or top to allow for hands free pumping?

- o Yes
- o No
- o Not applicable as I use / used a wearable or manual pump

47. How did you decide on which pump/s to use? Recommendation from: (Check all that apply)

- o Friends / Family
- o Midwife / OBGYN nurse
- o Online forum
- o Social media
- o Lactation consultant
- o Online reviews
- o Other (please state): ______

48. How did you get your breast pump/s?

- o Purchased during pregnancy
- o Purchased after birth
- o Hire
- o Gifted
- o Borrowed from friend / family
- o Other (please state): ______

49. What aspects of a breast pump are / were most important to you? (Check all that apply)

- o Quiet
- o Comfortable
- o Affordable price
- o Removes good volumes of milk
- o Removes milk fast

- o Easy to transport
- o Able to move around/use my hands when pumping
- o Other (please state): ______

50. What aspects of breast pumps need to be improved? _________

51. Where do / did you store your pumping kit between use?
- o In the refrigerator
- o In a container at room temperature
- o On the bench at room temperature
- o Wearable pump is / was left in my bra for two consecutive pumping sessions
- o Other: ________

52. How do / did you clean/sanitize your pumping equipment? (Check all that apply)
- o Rinse under water
- o Soap and water
- o Boiling pumping parts on the stove
- o Dishwasher
- o Microwave sterilizer
- o Steam sterilizer
- o Sterilizer solution (e.g Milton)
- o Other (please state): ______

53. How often do / did you clean your pumping equipment?
- o After each use
- o Once a day
- o Other: _______

Pumping Schedule

54. Do / did you usually double pump (pump both breasts at the same time) or single pump (pump one breast at a time)?
- o Single pump
- o Double pump
- o Alternate(d) between single and double pumping

55. How often do / did you typically pump in 24 hours? (Please count double pumping as one pumping session) _______

56. Do / did you pump at night (10pm – 4am)?
- o Yes
- o No (if No skip Q 56 and go to Q57)

57. How many times do / did you typically pump at night? _________

58. How long does / did each pumping session usually take (minutes)? ______ minutes

59. What is the minimum amount that you usually pump(ed) at a pumping session? (Total from both breasts) (Please state if in mL or oz) _______

60. What is the maximum amount that you usually pump(ed) at a pumping session? (Total from both breasts) (Please state if in mL or oz) __________

61. What is the total volume that you typically pump(ed) in 24 hours? (Please state if in mL or oz) _______

Feeding your baby

62. How much breast milk do / did you usually feed your baby per feeding? (Please state if in mL or oz) _______

63. How much breast milk do / did you usually feed your baby per 24 hours? (Please state if in mL or oz) ________

64. What information source/s guided you on how much milk to feed your baby? (Check all that apply)

- o Obstetrician / OBGYN
- o Pediatrician
- o Family physician / general practitioner
- o Midwife
- o Lactation consultant
- o Friends / family
- o Doula
- o Online forums
- o Social media
- o Websites
- o Child health nurse
- o Directed by baby

65. How do / did you decide when to feed your baby?

- o Feeding schedule
- o On demand
- o Other (please state): ______

_

66. How many times do / did you usually feed your baby in 24 hours? ______

67. Does / did your baby feed at night (10pm – 4am)?

- o Yes
- o No (If selected skip Q67, go to Q68)

68. If yes, how many times does / did your baby feed at night? _________

69. During your time of exclusive pumping what types of foods other than breast milk are / were fed to your baby? (Check all that apply)

- o Commercial milk formula (If selected answer Q69, 70 and 71)
- o Cow's milk
- o Other milk: soy milk, rice milk, goat milk, etc.
- o Solid foods (if selected answer Q72)

o Not applicable, I only feed / fed my baby expressed breast milk (If selected skip Qs 69-72, go to Q73)

70. How much formula do you usually feed / did feed your baby per feeding? (Please state if in mL or oz) _______

71. How much formula do you usually feed / did feed your baby per 24 hours? (Please state if in mL or oz) _______

72. At what age did you first introduce formula (weeks)? ________months

73. At what age did you first introduce solid foods (months)? _______ months

74. Who feeds / fed the baby your expressed milk?

o Mostly me
o Shared with my partner / support people
o Mostly my partner / support people

75. Who is / was responsible for cleaning your pumping kit?

o Mostly me
o Shared with my partner / support people
o Mostly my partner / support people

Milk Storage

76. Do you have any stored frozen expressed milk?

o Yes
o No (if No skip Q's 77 - 80, go to Q81)
o No, but I did freeze my expressed milk in the past

77. How much frozen expressed milk do you have? Or if you no longer pump what was the maximum amount of milk you had frozen? (Please state if in mL's, Ls or oz's) _________

78. What type of freezer do / did you use to store your breast milk?

o Freezer compartment of my refrigerator
o Chest / deep freezer
o Other: ________

79. What is the longest period of time you would store frozen milk for feeding your baby (months)? _________ months

80. What do / did you plan to do with your frozen breast milk after you stop(ped) pumping? (Check all that apply)

o Feed my baby when I have stopped pumping
o Supplement my fresh pumped milk
o Donate it
o Unsure
o Other (please describe): _______

Weaning

81. How old do / did you plan your child to be when stopping pumping (age in months)? _______ months

82. How old do you plan your child to be, or how old was your child when stopping feeding your breast milk (age in months)? _______months

Employment

83. How soon after giving birth will / did you return to paid work?

- o Less than 4 weeks
- o 4 – 12 weeks (1-3 months)
- o 12 – 24 weeks (3 – 6 months)
- o 6 months – 1 year
- o 1 + year
- o I will not be returning to work in the foreseeable future
- o Not applicable, I did not work before pregnancy

84. To what extent does / did returning to paid work influence your decision to exclusively pump for your baby?

- o Not applicable (not working / returning to work)
- o Very strongly influence
- o Strongly influences
- o Moderately influences
- o Slightly influences
- o No influence at all

Future babies

85. Do you plan on exclusively pumping for any future babies?

- o Yes, if the baby could not directly breastfeed
- o Yes, it is my preferred method of feeding
- o No
- o N/A, this is my last child

Thank you for participating in this study. Results of the study will be advertised on the Geddes Hartmann Human Lactation Research Group Facebook page after November 2025. You can follow the page here https://www.facebook.com/groups/humanlactationresearchgroup
